# Annual Wellness Visits and Timing of Advance Care Planning Among Medicare Beneficiaries with Cognitive Impairment

**DOI:** 10.1093/geroni/igaf122.3707

**Published:** 2025-12-31

**Authors:** Zhiwei Hu, Mukaila Raji, Yong Shan, Huey-Ming Tzeng, Sean O’Mahony, Yong-Fang Kuo

**Affiliations:** the University of Texas Medical Branch, Galveston, Texas, United States; University of Texas Medical Branch at Galveston, Galveston, Texas, United States; UTMB, Galvesto, Texas, United States; School of Nursing, University of Texas Medical Branch, Galveston, Galveston, Texas, United States; University of Texas Medical Branch, Galveston, Texas, United States; University of Texas Medical Branch, Galveston, Galveston, Texas, United States

## Abstract

Annual wellness visits (AWVs) offer an important opportunity to promote timely advance care planning (ACP) among older adults with mild cognitive impairment or dementia, but the real-world timing and equity of ACP initiation following AWVs remain poorly characterized. We conducted a retrospective cohort study of 959 405 fee-for-service Medicare beneficiaries aged 68 years or older newly diagnosed with cognitive impairment in 2018, using claims data through December 31, 2021, to examine whether AWV receipt was associated with earlier ACP initiation. Propensity‐score matching balanced sociodemographic, clinical, and utilization factors, and conditional Fine-Gray models accounting for death as a competing risk estimated hazard ratios. AWV receipt was associated with an 86% higher rate of first ACP use (HR, 1.86; 95% CI, 1.84–1.89). Cumulative incidence analyses showed that 9.0% of AWV recipients initiated ACP on the same day as their AWV, increasing modestly to 9.41% at 30 days, 14.78% at one year, and 27.62% at four years, whereas only 0.01% of non-AWV beneficiaries initiated ACP on their index date, reaching 17.46% by four years. Subgroup analyses showed the strongest effects among rural (HR, 3.34; 95% CI, 3.16–3.52) and Hispanic beneficiaries (HR, 2.78; 95% CI, 2.55–3.03). Sensitivity analyses censoring the switch of AWV between two groups or time-dependent AWVs yielded similar results. These findings indicate that AWVs serve as a key trigger for same-day ACP initiation and may help promote more timely and equitable ACP engagement in cognitively impaired older adults.

